# CD4 rate of increase is preferred to CD4 threshold for predicting outcomes among virologically suppressed HIV-infected adults on antiretroviral therapy

**DOI:** 10.1371/journal.pone.0227124

**Published:** 2020-01-06

**Authors:** Sol Aldrete, Jeong Hoon Jang, Kirk A. Easley, Jason Okulicz, Tian Dai, Yi No Chen, Maria Pino, Brian K. Agan, Ryan C. Maves, Mirko Paiardini, Vincent C. Marconi

**Affiliations:** 1 Division of Infectious Diseases, Medical College of Wisconsin, Milwaukee, Wisconsin, United States of America; 2 Department of Biostatistics and Bioinformatics, Rollins School of Public Health, Emory University, Atlanta, Georgia, United States of America; 3 Division of Internal Medicine and Infectious Disease Service, San Antonio Military Medical Center, San Antonio, Texas, United States of America; 4 Amgen Inc, Thousands Oaks, California, United States of America; 5 Department of Epidemiology, Emory University, Atlanta, Georgia, United States of America; 6 Division of Microbiology and Immunology, Yerkes Non-Human Primates Research Center and Emory Vaccine Center, Atlanta, Georgia, United States of America; 7 Department of Preventive Medicine and Biostatistics, Infectious Diseases Clinical Research Program, Uniformed Services University of the Health Sciences and Henry M. Jackson Foundation for the Advancement of Military Medicine, Rockville, Maryland, United States of America; 8 Division of Infectious Diseases, Naval Medical Center San Diego, San Diego, California, United States of America; 9 Division of Infectious Diseases, School of Medicine, Emory University, Atlanta, Georgia, United States of America; 10 Department of Global Health, Rollins School of Public Health, Emory University, Atlanta, Georgia, United States of America; 11 Atlanta Veterans Affairs Medical Center, Decatur, Georgia, United States of America; Katholieke Universiteit Leuven Rega Institute for Medical Research, BELGIUM

## Abstract

**Objectives:**

Immune non-responders (INR) have poor CD4 recovery and are associated with increased risk of serious events despite antiretroviral therapy (ART). A clinically relevant definition for INR is lacking.

**Methods:**

We conducted a retrospective analysis of three large cohorts: Infectious Disease Clinic at the Atlanta Veterans Affairs Medical Center, the US Military HIV Natural History Study and Infectious Disease Program of the Grady Health System in Atlanta, Georgia. Two-stage modeling and joint model (JM) approaches were used to evaluate the association between CD4 (or CD4/CD8 ratio) slope within two years since ART initiation and a composite endpoint (AIDS, serious non-AIDS events and death) after two years of ART. We compared the predictive capacity of four CD4 count metrics (estimated CD4 slope, estimated CD4/CD8 ratio slope during two years following ART initiation and CD4 at 1 and 2 years following ART initiation) using Cox regression models.

**Results:**

We included 2,422 patients. Mean CD4 slope (±standard error) during two years of ART was 102 ± 2 cells/μl/year (95% confidence interval: 98–106 cells/μl/year), this increase was uniform among the three cohorts (p = 0.80). There were 267 composite events after two years on ART. Using the JM approach, a CD4 slope ≥100 cells/μL/year or CD4/CD8 ratio slope >0.1 higher rate per year were associated with lower composite endpoint rates (adjusted hazard ratio [HR] = 0.80, p = 0.04 and HR = 0.75 p<0.01, respectively). All four CD4 metrics showed modest predictive capacity.

**Conclusions:**

Using a complex JM approach, CD4 slope and CD4/CD8 ratio slope the first two years after ART initiation were associated with lower rates of the composite outcome. Moreover, the uniformity observed in the mean CD4 slope regardless of the cohort suggests a common CD4 response pattern independent of age or CD4 nadir. Given the consistency observed with CD4 slope, availability and ease of interpretation, this study provides strong rationale for using CD4 gains <100 cells/μl/year to identify patients at risk for adverse events.

## Introduction

The development of antiretroviral therapy (ART) has significantly changed the survival and prognosis of people living with HIV [1]. Most individuals who initiate ART experience sustained increases in their peripheral CD4^+^ T cell (CD4) counts, the hallmark of clinical recovery. Several studies have noted that there is a significant proportion of individuals who fail to restore their CD4 counts despite virologic suppression on ART for several years [2, 3]. In the literature, these individuals are labeled as discordant immune responders or immune non-responders (INR). Multiple factors have been associated with INR (i.e. older age, low CD4 nadir, long duration of HIV infection) but none of these factors provides a full explanation for the lack of immune reconstitution [4]. Additionally, INR have been associated with an increased risk of AIDS and serious non-AIDS events as well as mortality [5–7]. For these reasons, substantial efforts are being pursued to improve the identification and management of this key population [7–10].

There is no consensus in the literature on how best to define INR [7, 9]. Some studies define INR as a failure to achieve a pre-specified absolute CD4 count (threshold) at a particular time point [11–13] while others require a pre-specified increase in CD4 count irrespective of the initial value [14–17]. Most studies focus on a threshold (i.e. <200 or <350 cells/μl) derived from prior guidelines that determined timing of ART initiation and risk of opportunistic infections based on CD4 counts. Two studies even found a comparable morbidity and mortality rate to the general population for patients that achieve a CD4 >500 cells/μl [18, 19]. Recently, increasing emphasis has been placed on the CD4/CD8 ratio, as low values have been associated with increased immune activation and immunosenescence, which in turn have been associated with increased morbidity and mortality for people living with HIV [20, 21]. When examining the predictive capacity of these measures with long-term outcomes, there have been conflicting results given the diversity of populations studied, inclusion and exclusion criteria employed, and outcomes analyzed [7, 10].

In an effort to characterize clinical outcomes associated with INR, several analytic approaches have been used to quantify the relationship between longitudinal CD4 count data and time to event data. One approach has been to employ Cox proportional hazards regression models with time-dependent covariates (i.e. CD4 counts). However, standard statistical models such as the linear mixed model for longitudinal data and the Cox proportional hazards model for time-to-event data do not consider dependencies between these two different data types. Alternative approaches have been suggested in the biostatistics literature, such as two-stage modeling and joint modeling (JM) analyses, which enable both longitudinal repeated biomarker measurements and survival processes to be modeled together while accounting for the association between them, which leads to enhanced efficiency and correction of biases [22, 23].

In light of the clinical relevance of INR, the wide range of definitions available in the literature and the standard statistical models used to examine the association between CD4 counts and outcomes, the objective of this analysis was two-fold. The first aim was to identify a universal CD4 count or CD4/CD8 ratio recovery pattern most predictive of composite adverse events after two years since ART initiation across three diverse patient populations using a two-stage modeling and JM approach. The second aim was to assess and compare the predictive capacity of Cox regression models based on four continuous CD4 count metrics: CD4 counts at one year and at two years after initiating ART, estimated CD4 count slope and estimated CD4/CD8 ratio slope during the two years following ART initiation.

## Methods

### Study setting and population

We utilized data from three large, previously well-described clinical cohorts with distinct patient characteristics: the Infectious Disease Clinic at the Atlanta Veterans Affairs (VA) Medical Center (AVAMC) which is the largest HIV clinic in the VA [24, 25], the US Military HIV Natural History Study (NHS) [26] and the Infectious Disease Program (IDP) of the Grady Health System in Atlanta, Georgia which is one of the largest HIV clinics in the United States [27].

Patients were eligible for this study if they were ART naïve upon entry into the clinical cohort. Additionally, all patients had to achieve virologic suppression (VL <1,000 copies/μl) at six months or earlier after initiating ART and remain suppressed for at least the first two years after initiating ART. This VL threshold was chosen to overcome the heterogeneity of the assay detection limit used to quantify RNA among the distinct cohorts and eras (all patients started ART after 1995). Other demographic and clinical variables obtained included gender, race, age at baseline, VL measurements and nadir CD4 counts. Study follow-up time was censored after 12 years from the date of ART initiation.

### Definitions

Length of follow up was defined from ART start to the latest CD4 measurement available for each patient. The composite endpoint of interest was the first occurrence of any of the following events after two years of ART initiation and before censoring: an acute cardiovascular disease event (myocardial infarction, acute transient ischemic attack/stroke, cardiac arrest, acute congestive heart failure), an AIDS-defining illness (excluding a CD4 <200), malignancy (excluding benign tumors or skin cancer), end-stage renal disease requiring hemodialysis, liver disease (acute liver failure or cirrhosis), significant infections (bacterial pneumonia, bacterial meningitis, endocarditis or influenza), pulmonary disease (COPD) or death. For the AVAMC and IDP, outcomes were abstracted from electronic health registry data using International Classification of Diseases-9 (ICD-9) codes. For the NHS, the clinical diagnoses were mapped onto equivalent ICD-9 codes.

### Institutional Review Board approval

The NHS cohort has been approved by the Institutional Review Board (IRB) centrally (Uniformed Services University of the Health Sciences, Bethesda, MD) and at each participating center (Walter Reed National Military Medical Center, Bethesda, MD; Naval Medical Center, Portsmouth, VA; San Antonio Military Medical Center, TX; Naval Medical Center, San Diego, CA; and Tripler Army Medical Center, Honolulu, HI). Written consent was obtained from each patient. The AVAMC cohort involved a secondary analysis of electronic health record (EHR) data that was approved by the Emory University IRB and that Atlanta VA Medical Center Research and Development Committee. The AVAMC analysis had an IRB-approved HIPAA waiver and therefore did not require informed consent. The IDP cohort was also a secondary analysis of data from the Center for AIDS Research (CFAR) HIV Data Registry within the Grady Health System. The Registry contains EHR data from patients who received medical treatment at the Ponce de Leon Center IDP. This analysis also has an IRB-approved HIPAA waiver and did not require informed consent.

### Statistical analysis

Patient demographic and clinical characteristics were compared between the cohort groups with the one-way analysis of variance (ANOVA) F-test for continuous variables and the chi-square test for categorical variables. Cumulative composite rates were estimated using the Kaplan-Meier method and composite rates between immune responders and INR were compared by the log-rank test. Hazard ratios were calculated to measure the degree of association between the demographic and baseline clinical characteristics and the composite outcome by fitting the Cox proportional-hazards regression model separately for each baseline characteristic.

#### Association analyses via CD4 modeling

The primary predictor of interest was the CD4 count slope during the two years following ART initiation. Two approaches were used to evaluate the association between the CD4 count slope and the composite endpoint, two-stage modeling and JM. Details about the statistical methods used are provided in the S1 Appendix. The unadjusted hazard ratios for the composite endpoint were summarized per 50 or 100 cells/μL/year increase in CD4 slopes and per 50 or 100 cells/μL increase in CD4 intercepts for ease of interpretation of the regression coefficients. Adjusted hazard ratios (HR) and their 95% confidence intervals were obtained using a multivariable Cox model including gender, race, baseline age group (≤ 37 or >37 at baseline), study cohort and estimated CD4 slope and intercept.

We also examined the relationship between the composite outcome rate and the CD4 recovery status, as indicated by a slower or faster rate of CD4 count increase in the first two years after ART initiation. Specifically, the estimated CD4 slopes during the two years following ART initiation from the linear mixed-effects model were dichotomized at the median to categorize patients as immune non-responders (INR ≤100 cells/μL/year) or immune responders (IR >100 cells/μL/year), and then the cumulative incidence of the composite outcome by the CD4 recovery status was calculated. Next, using the CD4 recovery status as a binary predictor variable and the composite outcome as a clinical endpoint, we conducted Cox proportional hazards regression analyses, including adjustment for gender, race, baseline age group and estimated CD4 intercept. The degree of effect modification by age was visualized by plotting the 10-year cumulative incidence smoothed over the entire age range and stratified by the CD4 recovery status.

#### Association analyses via CD4/CD8 ratio modeling

Another predictor of interest was CD4/CD8 ratio slope during the two years following ART initiation. The two aforementioned approaches, two-stage modeling and JM were repeatedly adopted to evaluate the association between the CD4/CD8 ratio slope and the composite endpoint. See S1 Appendix for more details.

#### Comparison of CD4 count metrics

This analysis focused on assessing and comparing the predictive ability of the Cox regression models based on four continuous CD4 count metrics: (i) estimated CD4 slope during the two years following ART initiation (two-stage model approach); (ii) estimated CD4/CD8 ratio slope during the two years following ART initiation (two-stage model approach); (iii) CD4 cell counts at 1 year after initiating ART; and (iv) CD4 cell counts at 2 years after initiating ART. For the two-latter metrics, CD4 counts closest to the first and second year of ART were used, respectively. The Cox models were also further adjusted for study cohort, age at baseline and gender in subsequent analyses. Time-dependent receiver operating characteristic (ROC) curves, area under the time-dependent ROC curves (AUC) and Uno’s C-statistics were used to assess and compare the discriminative performance of the final prediction model. All three statistics were appropriate for right-censored data.

All statistical analyses were conducted using SAS software version 9.4 (SAS Institute Inc, Cary, NC). Statistical significance was evaluated at a type I error type of 0.05. All statistical tests were 2-sided. JM were fit using a SAS macro JMFit [28]. The S2 Appendix provides SAS code for conducting two-stage modeling and JM analyses.

## Results

### Patient characteristics

The combined study cohort included 2,422 ART naïve patients who became virologically suppressed after ART initiation and remained suppressed for at least two years (Table 1). The mean age at ART initiation was 37.6 years, most were male (87%) and African-American (62%). At the start of ART, mean CD4 count was 244 cells/μL (standard deviation [SD] = 208) and mean CD4 nadir was 208 cells/μL (SD = 177). Median follow up was 5.4 years for patients without the composite endpoint (interquartile range [IQR], 3.4–7.9 years). The three cohort groups differed significantly in age at baseline, ethnicity, CD4 nadir and CD4 count at baseline. There were 267 composite endpoints after two years on ART. 73 (27.3%) were AIDS-related events, 67 (25%) were related to infections, 47 (17.6%) were cancer, 42 (15.7%) were cardiovascular events, 16 (6%) related to liver events, 10 (3.7%) pulmonary events, 3 (1.1%) renal events and 9 deaths.

**Table 1:** Demographic and clinical characteristics by cohort.

	All Cohorts	AVAMC	IDP	NHS	P-value
**N (%)**	2422	262 (11)	1146 (47)	1014 (42)	
Age at ART initiation, Mean (SD)	37.6 (10.8)	45.7 (10.9)	39.8 (10.5)	33.1 (9.07)	< 0.001^a^
Gender (%)					< 0.001^b^
Female	315 (13)	4 (2)	260 (23)	51 (5)	
Male	2107 (87)	258 (98)	886 (77)	963 (95)	
Race/Ethnicity^d^ (%)					< 0.001^b^
Caucasian	602 (25)	41 (16)	150 (13)	411 (41)	
African American	1492 (62)	212 (81)	864 (75)	416 (41)	
Hispanic	208 (9)	0 (0)	78 (7)	130 (13)	
Other	114 (5)	9 (3)	54 (5)	51 (5)	
CD4 count at baseline (cell/μL), mean (SD)	244 (208)	276 (197)	108 (111)	388 (194)	< 0.001^a^
CD4 nadir (cell/μL), mean (SD)	208 (177)	222 (166)	90 (93)	337 (159)	< 0.001^a^
Baseline viral load (copies/μL), median (IQR)	47,863 (10902–134015)	61,636 (16074–177138)	60,255 (13489–181970)	37,914 (7561–100000)	< 0.001^c^
Years of follow-up after baseline, median (IQR)	5.4 (3.4–7.9)	6.3 (4.0–10.1)	4.7 (3.2–6.7)	6.0 (3.7–9.7)	< 0.001^c^
Total number of CD4 measurements (%)	16888	2025 (12)	8481 (50)	6382 (38)	
Number of CD4 measurements during the two years following ART initiation, median (IQR)	7 (5–8)	7 (5–9)	7 (6–8)	6 (4–8)	< 0.001^c^
Total number of CD4/CD8 ratio measurements (%)^e^	16560	2025 (12)	8155 (49)	6380 (39)	
Number of CD4/CD8 ratio measurements during the two years following ART initiation, median (IQR)	7 (5–8)	7 (5–9)	7 (6–8)	6 (4–8)	< 0.001^c^

Abbreviations: AVAMC = Infectious Disease Clinic at the Atlanta Veterans Affairs (VA) Medical Center. IDP = Infectious Disease Program of the Grady Health System. NHS = US Military HIV Natural History Study. SD, standard deviation; IQR, interquartile range.

^a^One-way analysis of variance (ANOVA) F-test.

^b^Chi-square test of independence.

^c^Kruskal–Wallis test.

^d^6 observations were missing for race.

^e^CD4/CD8 ratio measurements were collected from 2380 patients (out of 2422 patients)

### CD4 count and CD4/CD8 ratio characteristics

The estimated CD4 slope (mean ± standard error [SE]) was 102 ± 2 cells/μL/year. The mean CD4 count at one year and at two years was 427 cells/μL and 530 cells/μL, respectively. The mean CD4 intercept, mean CD4 count at year 1 and at year 2 were significantly different between the three cohorts, but there were no differences in the mean CD4 slopes (p = 0.80; Table 2; S1A Fig). The estimated CD4/CD8 ratio slope (mean± SE) was 0.169 ± 0.003. The mean CD4/CD8 ratio intercept, slope and ratio at one and two years were significantly different between the three cohorts (S1 Table and S1D Fig).

**Table 2:** Estimated^a^ mean CD4 intercept (cell/μL), estimated mean CD4 count (cell/μL) after 1 and 2 years from ART initiation and the estimated mean CD4 slope (cell/μL/year) during the two years following ART initiation by cohort, CD4 recovery status and baseline age group.

	Mean CD4 Intercept (SE)	Mean CD4 slope (SE)	Mean CD4 Count at Year 1 (SE)	Mean CD4 Count at Year 2 (SE)
All	326 (5)	102 (2)	427 (5)	530 (6)
Cohort^b^				
AVAMC	361 (11)	102 (6)	463 (11)	565 (14)
IDP	180 (5)	103 (3)	283 (6)	386 (7)
NHS	482 (6)	100 (3)	582 (6)	682 (7)
P-value^g^	< 0.0001	0.8044	< 0.0001	< 0.0001
CD4 recovery status^c^				
Non-responders	326 (6)	38 (2)	363 (6)	401 (6)
Responders	327 (7)	178 (2)	505 (7)	683 (7)
P-value^g^	0.8742	< 0.0001	< 0.0001	< 0.0001
Baseline age group^d^				
Age > 37	269 (6)	99 (3)	368 (7)	467 (8)
Age ≤37	377 (7)	104 (3)	482 (7)	585 (8)
P-value^g^	< 0.0001	0.1843	< 0.0001	< 0.0001
Race^e^				
Caucasian	411 (10)	98 (4)	509 (10)	607 (12)
African-american	284 (6)	104 (2)	388 (6)	492 (6)
Hispanic	372 (17)	100 (6)	471 (17)	572 (19)
Other/not specified	317 (22)	105 (9)	421 (24)	526 (29)
P-value^g^	< 0.0001	0.7048	< 0.0001	< 0.0001
Gender^f^				
Female	233 (11)	127 (5)	360 (10)	487 (13)
Male	339 (5)	98 (2)	437 (5)	536 (6)
P-value^g^	< 0.0001	< 0.0001	< 0.0001	< 0.0001

Abbreviations: AVAMC = Infectious Disease Clinic at the Atlanta Veterans Affairs (VA) Medical Center. IDP = Infectious Disease Program of the Grady Health System. NHS = US Military HIV Natural History Study. SE, standard error

^a^Estimated from linear mixed-effects model specifying that CD4 cell counts during the two years following ART initiation follow a linear model regression over time, with a random intercept and slope for each patient.

^b^Additional categorical covariate representing the study cohort is included the linear mixed-effects model.

^c^Additional binary covariate representing the immune status is included the linear mixed-effects model.

^d^Additional binary covariate representing the baseline age group is included the linear mixed-effects model.

^e^Additional categorical covariate representing the race group is included the linear mixed-effects model.

^f^Additional binary covariate representing the gender is included the linear mixed-effects model.

^g^Result of a likelihood ratio test comparing estimated CD4 intercepts, counts at years 1 and 2, and CD4 slopes among different categories of a given covariate

When comparing IR to INR (≤100 cells/μL/year) based on our *a priori* definition, the mean CD4 slope was significantly different (IR 178 ±2 vs. INR 38 ±2 cells/μL/year, P <0.0001) despite starting at the same mean CD4 intercept (P = 0.87; S1B Fig). For the ratio, the mean CD4/CD8 intercept and slope were significantly different between IR and INR (S1E Fig).

### CD4 modeling

For the two-stage model approach, the unadjusted analysis showed a significant association between CD4 slope in the first two years of ART and the composite endpoint (P = 0.02; Table 3). This effect was maintained for the adjusted analysis (P = 0.04; Table 3). Likewise, under the JM approach, a higher CD4 slope in the first two years of ART was associated with a lower likelihood of developing the composite endpoint in the unadjusted analysis (P = 0.02; Table 3). This effect was also observed in the adjusted analysis for the 50 cells/μL/year increase (HR = 0.90, 95% CI: 0.81–0.99, P = 0.04), or equivalently for the 100 cells/μL/year increase (HR = 0.80, 95% CI: 0.65–0.99, P = 0.04; Table 3). For both approaches, CD4 intercept exhibited a highly significant association with the composite outcome in the unadjusted analysis (P <0.01) but did not show an association in the adjusted analyses. The hazard ratios were very similar in a sensitivity analysis of the subset of 1,837 patients who remains virologically suppressed (VL <1,000 copies/μl) throughout their follow up.

**Table 3:** Association between estimated CD4 intercept and CD4 slope during the two years following ART initiation with risk of composite endpoint^a^.

	Unadjusted^b^			Adjusted^c^		
	HR	95% CI	P-value	HR	95% CI	P-value
**Two-Stage Model^a^**						
Estimated CD4 Intercept			0.004			0.0821
Per 50 increase in cell/μL	0.94	0.91–0.98		0.97	0.93–1.01	
Per 100 increase in cell/μL	0.89	0.84–0.95		0.94	0.87–1.01	
Regression coefficient	$\hat{\beta }$ (SE) = -0.0012 (0.0003)			$\hat{\beta }$ (SE) = -0.0007 (0.0004)		
Estimated CD4 Slope			0.0190			0.0371
50 cell/μL/year increase	0.88	0.79–0.98		0.89	0.80–0.99	
100 cell/μL/year increase	0.78	0.63–0.96		0.80	0.65–0.99	
Regression coefficient	$\hat{\beta }$ (SE) = -0.0025 (0.0011)			$\hat{\beta }$ (SE) = -0.0023 (0.0011)		
**Joint Model^d^**						
Estimated CD4 Intercept			0.0019			0.0615
Per 50 increase in cell/μL	0.95	0.92–0.98		0.96	0.93–1.00	
Per 100 increase in cell/μL	0.90	0.85–0.96		0.93	0.86–1.00	
Regression coefficient	$\hat{\beta }$ (SE) = -0.0010 (0.0003)			$\hat{\beta }$ (SE) = -0.0007 (0.0004)		
Estimated CD4 Slope			0.0212			0.0436
50 cell/μL/year increase	0.88	0.80–0.98		0.90	0.81–0.99	
100 cell/μL/year increase	0.78	0.63–0.96		0.80	0.65–0.99	
Regression coefficient	$\hat{\beta }$ (SE) = -0.0024 (0.0011)			$\hat{\beta }$ (SE) = -0.0022 (0.0011)		

Abbreviations: CI = confidence interval. HR = hazard ratio.

^a^The risk of composite endpoint was modeled using a Cox proportional hazards regression model considering estimated CD4 slope and intercept obtained from the linear mixed-effects model as continuous covariates.

^b^Results obtained from Cox submodels for the composite outcome that include estimated CD4 slope and intercept as covariates.

^c^Results obtained from the Cox submodels for the composite outcome that include estimated CD4 slope and intercept, study cohort, baseline age group (≤ 37 or >37 at baseline) and gender as covariates.

^d^The risk of composite endpoint was modeled using the joint model that enables both longitudinal CD4 measurements and clinical endpoint data to be modelled together while taking account for the interrelationship between the two components

When evaluating the association between CD4/CD8 ratio slope in the first two years of ART with the composite endpoint, the two-stage model approach showed a significant association with 0.1 or 0.2 higher rate per year increase (P <0.01). This effect was maintained for the adjusted analysis of both CD4/CD8 ratio increases. In the JM approach, a 0.1 and 0.2 higher rate per year increase were significant in the unadjusted and adjusted analyses (S2 Table).

**Fig 1:**
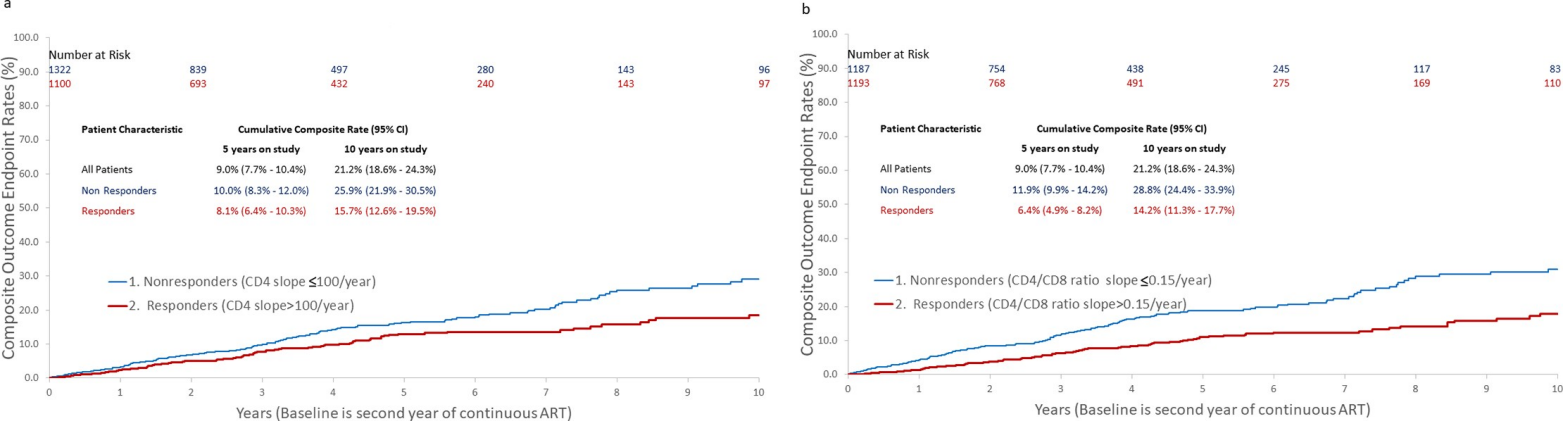
**(a)** Cumulative composite outcome endpoint rates by CD4 recovery status. Responders had CD4 slope >100 cells/μL/year. Estimated with the Kaplan-Meier method and compared by the log-rank test (p < 0·01). The median follow-up time estimated by a reverse Kaplan-Meier method was 5.5 years (95% CI: 5.3–5.6). **(b)** Cumulative composite outcome endpoint rates by CD4/CD8 recovery status. Responders had CD4/CD8 ratio slope >0.15 per year. Estimated with the Kaplan-Meier method and compared by the log-rank test (p < 0·01). The median follow-up time estimated by a reverse Kaplan-Meier method was 5.5 years (95% CI: 5.3–5.6).

The 10-year cumulative incidence rate of the composite endpoint was 15.7% (95% CI: 12.6%-19.5%) for IR versus 25.9% (95% CI: 21.9%-30.5%) for INR (P <0.01; Fig 1A). For the CD4/CD8 ratio, IR (CD4/CD8 ratio slope >0.15 per year) had a composite outcome of 14.2% (95% CI: 11.3%-17.7%) versus 28.8% (95% CI: 24.4%-33.9%) for INR (Fig 1B).

Effect modification analysis suggests a difference across levels of age for both CD4 slope and CD4/CD8 ratio slope (P = 0.07 and 0.03 for the statistical interaction between age and immune response by CD4 slope and CD4/CD8 ratio slope, respectively). For the CD4 slope, the composite endpoint rate decreased 39% among older IR (above the median age of 37 years) compared to older INR (HR = 0.61, 95% CI: 0.44–0.84, P = 0.003, S3 Table). This effect was not seen in the younger population (S3 Table). A similar effect modification was observed for the CD4/CD8 ratio slope (S4 Table).

**Fig 2:**
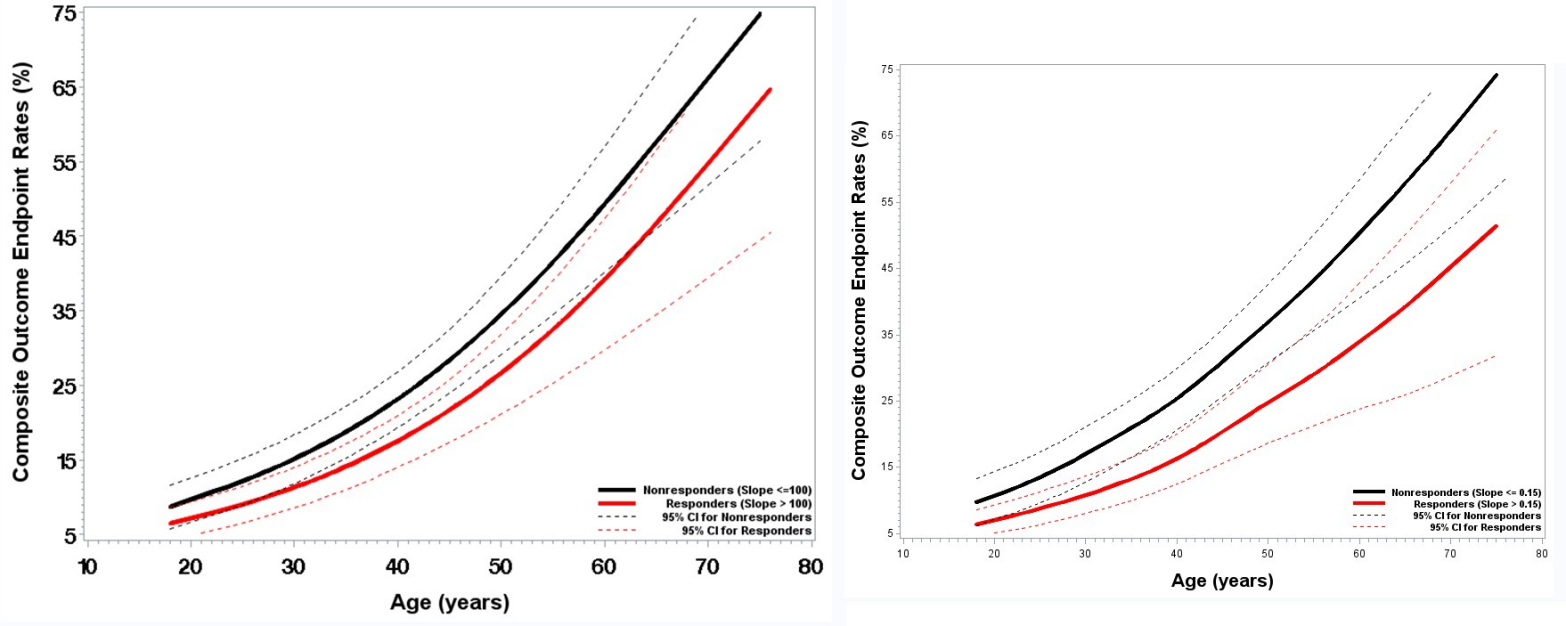
Smoothed cumulative composite outcome endpoint rate curves and their 95% confidence intervals at 10 years from ART initiation by age and immune status (2a: CD4 recovery status; 2b: CD4/CD8 ratio recovery status).

Fig 2A and 2B provides smoothed 10-year cumulative composite rate curves for IR and INR (CD4 slope and CD4/CD8 ratio) by baseline age. The cumulative incident rates were lower for IR at all ages with increasing discrepancy between the two groups over age.

### CD4 metrics comparison

**Fig 3:**
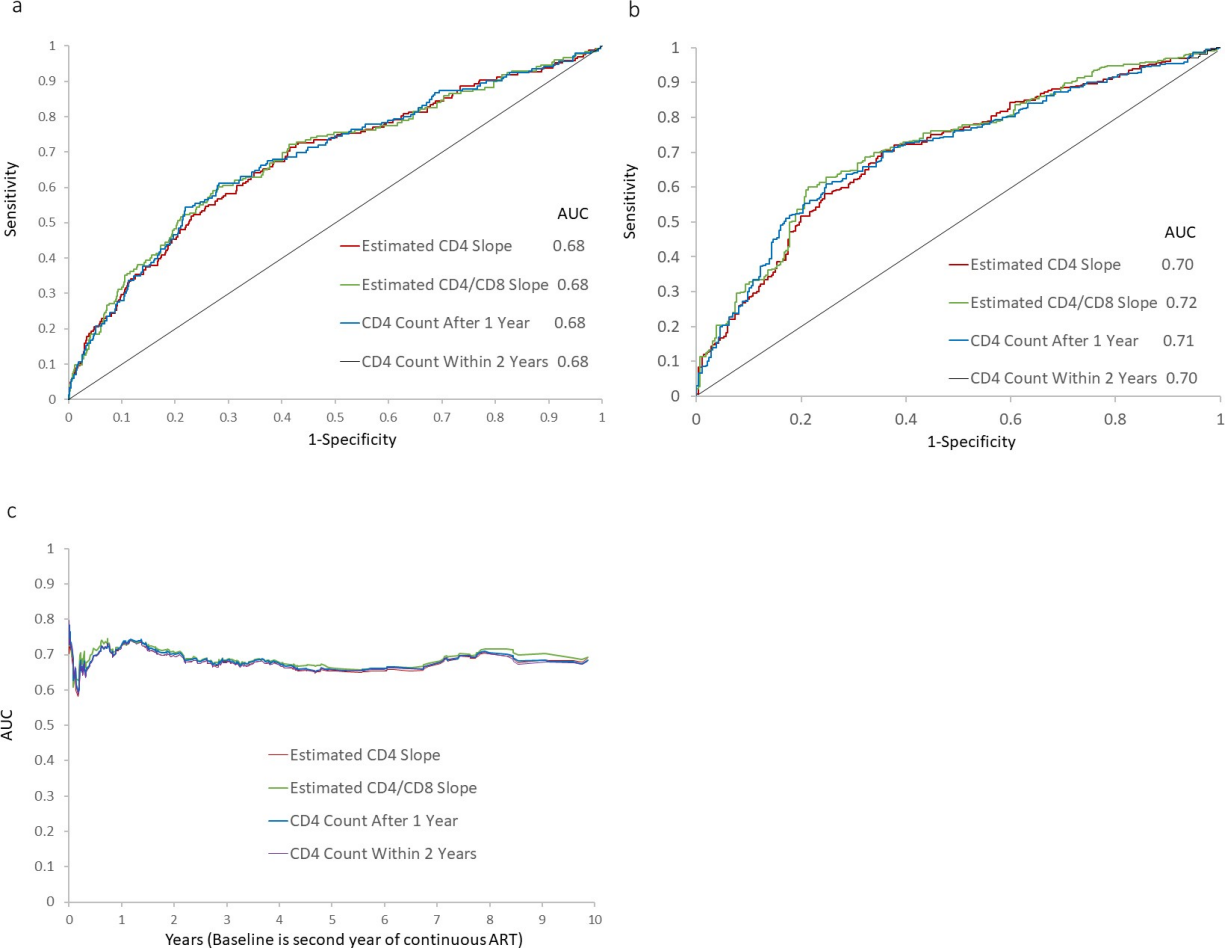
Time-dependent ROC curves at (3a) 5 and (3b) 10 years from ART initiation and (3c) time-dependent AUCs evaluated over the 10-year study period (baseline at second year of continuous ART). Cox regression models include (i) Estimated CD4 slope (ii) Estimated CD4/CD8 ratio slope (iii) CD4 counts after 1 year and (iv) CD4 counts after 2 years as main predictors, respectively, and adjusted for estimated CD4 intercept or estimated CD4/CD8 ratio or baseline CD4 count, depending on the choice of the prognostic risk factor, and commonly adjusted for age at baseline, gender and study cohort.

Finally, we evaluated the predictive accuracy of the Cox regression models for the composite endpoint employing the four continuous CD4 metrics previously mentioned. For the four prognostic factors, the 5-year and 10-year (S2A and S2B Fig) AUC values ranged from 0.59 to 0.63 and 0.66 to 0.70, respectively, with those of the estimated CD4/CD8 ratio slope being the highest at both time points. The time-dependent AUC curves for the four prognostic factors exhibited nearly identical patterns, hovering above 0.65 at earlier and later time points, but falling to about 0.6 in between, while that of the estimated CD4/CD8 ratio slope attained highest values over most of the study period (S2C Fig). In terms of the Uno’s C-statistic, the estimated CD4/CD8 ratio slope appeared to provide significantly higher prognostic information compared to other prognostic factors (S5 Table). Prediction of the composite endpoint generally improved and was more similar among the four prognostic factors using the adjusted Cox regression models. For each of the prognostic factors, both the 5-year and 10-year AUC values were approximately 0.70 (Fig 3A and 3B), and the time-dependent AUC curve hovered around 0.70 over most of the study period (Fig 3C), suggesting acceptable (fair) predictive accuracy of the composite endpoint with the Cox regression models. Uno’s C-statistics were between 0.64 and 0.66 without any significant difference among the prognostic factors (S5 Table).

## Discussion

Considering the significant morbidity and mortality associated with INR, we were interested in identifying the CD4 count and CD4/CD8 ratio recovery pattern most predictive of long-term outcomes in HIV patients who started ART and remained suppressed for the first two years. In our combined study cohort, we found that both CD4 slope (>100 cells/μl) and CD4/CD8 ratio slope (>0.1 higher per year) during the first two years after ART initiation were associated with lower rates of the composite endpoint. Prior studies have found similar results, a CD4 slope <100 cells/μl has been used to define INR in association with developing new AIDS defining illness [16] or mortality [14] and other studies have equally reported on the inverse relationship between the CD4/CD8 ratio and risk of cardiovascular events [29], serious non-AIDS events and deaths[30]. However, we believe our study offers an advantage to prior statistical analysis performed in that we used a complex modeling approach to explore the longitudinal relationship between CD4 counts and composite clinical events which allows to consider the dependencies between these two different data types and reduces substantial bias that emerges with other biostatistical approaches. To our knowledge, this is the first study in the literature using a JM approach to explore the relationship of CD4 recovery and composite clinical events. All the studies published to date have been limited to biostatistical modeling without providing clinical endpoints [22, 31–33].

We also found that age at ART initiation had a significant effect on both the immune response for CD4 slope and CD4/CD8 ratio slope. The effect of age on immune recovery has been previously described [2, 21], and it is well known that younger age is associated with higher absolute CD4 cell gain and shorter time to a maximum response independent of baseline CD4 [34]. Age has also been found to be an independent predictor of long-term outcomes [35]. In our study, we found a marked difference in the association between the immune recovery and risk of the composite endpoint based on age (>37 years old). The fact that this effect was not observed in younger INR could be due to either lower levels of immune activation or presenting for care soon after infection.

Given the wide range of INR definitions in the literature (S3 Appendix) and the challenges associated with performing a meta-analysis, our second aim was to compare the most common CD4 metrics used in the literature. In our case, a direct comparison by cutpoints created by dichotomizing continuous prognostic factors such as CD4 count was not attempted due to several serious drawbacks: loss of power and precision due to data loss, “optimal” cut points do not replicate across studies; and cut points are arbitrary and can be manipulated [36, 37]. In contrast to other studies which have looked at the prognostic role of various specifications of CD4 recovery and outcomes via cox regression models and hazard ratios [35, 38], we examined time-dependent ROC curves, time-dependent AUCs and Uno’s C-statistics to assess and compare the discriminative performance of the final prediction model. All four CD4 metrics were modest predictors of the composite outcome. Although there was a trend for the CD4/CD8 ratio to do slightly better, formally comparing the difference between all pairwise AUCs at 5 and 10 years provided evidence that none of the markers were superior as a predictor of the composite endpoint in the unadjusted and adjusted analysis.

Recent experts have recommended less frequent CD4 monitoring in virologically suppressed individuals who start therapy at high CD4 counts[39]. However, current DHHS guidelines still recommend regular CD4 monitoring (i.e. every 3–6 months) during the first two years of ART and then annually if CD4 remains below 500 cells/μl[8]. Our study supports the importance of monitoring the initial CD4 response within the first two years after ART initiation and provides validity for these results even in patients starting with high CD4 counts (i.e. NHS cohort).

Study strengths included a multicenter study of three different cohorts which differed in age, baseline CD4 count and time to ART initiation allowing for a broader application of our results. Additionally, this analysis included a composite endpoint and not just mortality. A sub-analysis excluding the pulmonary outcomes and influenza diagnoses yielded similar results. In addition, the JM approach, although complex and labor intensive, decreased the potential for bias and allowed for simultaneous modeling of both the repeated CD4 count measurements and clinical endpoint [33].

Our study has several limitations. First, our analysis was retrospective with two of the cohorts utilizing clinical observational data. Second, there could have been residual confounding from unmeasured patient characteristics that affected the CD4 response (i.e. seroconversion date, hepatitis B or C infection, ART). Third, we used an expansive definition for viral suppression, allowing patients to remain in the analysis if their plasma HIV viral load levels remained <1000 copies/μl although this definition has been used by other studies[3, 6, 17]. A sub-analysis for the patients with a viral load ≤400 or ≤200 copies/μl reduced the population being evaluated but gave results with a similar trend. An additional sub-analysis on those patients who remained virologically suppressed (VL <1000 copies/μl) through out their follow up showed a similar trend.

When looking at the JM analysis, our study found that both CD4 slope and CD4/CD8 ratio slope were associated with lower rates of the composite outcome. However, we believe CD4 slope is more practical to use in a clinical setting as it is more readily available and easy to interpret. Moreover, the mean CD4 slope was the same in each cohort while the starting and final CD4 counts and CD4/CD8 ratio were different. These findings suggest a common CD4 response pattern among patients, independent of age, race and CD4 at ART initiation. By using the rate to identify INR, instead of a threshold, we were able to include populations with different clinical characteristics allowing for a more widespread application of our findings. In addition, this might also suggest that patients who have a high CD4 baseline are still at risk of adverse clinical events if their CD4 counts do not increase appropriately after starting ART. In conclusion, an average CD4 count increase >100 cells/μL/year predicts better clinical outcomes. Among those with CD4 gains ≤100, increased monitoring for adverse clinical events may be warranted. The next steps will be to incorporate additional variables into these models to enhance their predictive accuracy.

## Supporting information

S1 Appendix Supplementary methods. (DOCX)

S2 Appendix SAS v9.4 code for conducting two-stage modeling and joint modeling analyses. (DOCX)

S3 Appendix INR definitions in the literature. (DOCX)

S1 Table Estimateda mean CD4/CD8 ratio intercept, estimated mean CD4/CD8 ratio after 1 and 2 years from ART initiation and the estimated mean CD4/CD8 ratio slope (per year) during the two years following ART initiation with treatment interruptions of less than 2 months by cohort, CD4/CD8 ratio recovery status and baseline age group. (DOCX)

S2 Table Association of estimated CD4/CD8 ratio slope and intercept during the two years following ART initiation with risk of composite endpoint. (DOCX)

S3 Table Association between patient CD4 recovery status based on estimated CD4 slope and risk of composite endpoint by baseline age group, gender and race. (DOCX)

S4 Table Association between patient CD4/CD8 ratio recovery status based on estimated CD4/CD8 ratio slope and risk of composite endpoint by baseline age group, gender and race. (DOCX)

S5 Table Comparison of Uno’s C-statistics among the four prognostic risk factors: (i) Estimated CD4 slope (ii) Estimated CD4/CD8 ratio slope (iii) CD4 counts after 1 year and (iv) CD4 counts after 2 years. (DOCX)

S1 Fig **Estimated mean CD4 cell counts (cells/ μL) (left panel) and estimated mean CD4/CD8 ratio (right panel) at baseline and after 1 and 2 years in the first 2 years on continuous ART for at least 24 months by study cohort (S1a, S1d), immune status (S1b, S1e) and age at baseline (S1c, S1f).** Vertical bars are 95% confidence intervals.
(TIF)

S2 Fig **(a)** CD4 metrics time-dependent ROC curves at 5 years from ART initiation. **(b)** CD4 metrics time-dependent ROC curves at 10 years from ART initiation. **(c)** CD4 metrics time-dependent AUCs evaluated over the 10-year study period.
(TIF)
