# Supplementary material for: CD4 rate of increase is preferred to CD4 threshold for predicting outcomes among virologically suppressed HIV-infected adults on antiretroviral therapy
Source: PLoS One. 2020 Jan 6;15(1):e0227124. doi: 10.1371/journal.pone.0227124 (PMC6944336; doi:10.1371/journal.pone.0227124)
Supplement: S1 Appendix — (DOCX) [file pone.0227124.s001.docx]

**S1 Appendix**

**Supplementary Methods**

**Association analyses via CD4 modeling.**

Two approaches were used to evaluate the association between the CD4 count slope and the composite outcome. First, two-stage modeling was used to control for covariate confounding. In the first stage, CD4 count slopes and intercepts were estimated using a mixed-effects model specifying that CD4 counts during the two years following ART initiation follow a linear regression over time with a random slope and intercept for each patient. Specifically, the patient-specific coefficient deviation (the random effect) was added to the overall coefficient (the fixed effect) to obtain the patient-specific intercept and slope for each patient. In the second stage, the Cox proportional hazards regression model was fit for the composite outcome using both patient-specific CD4 slope and intercept estimates obtained from the mixed-effects model as continuous covariates. The unadjusted hazard ratios for the composite endpoint were summarized per 50 or 100 cells/µL/year increase in CD4 slopes and per 50 or 100 cells/µL increase in CD4 intercepts for ease of interpretation of the regression coefficients. Adjusted hazard ratios and their 95% confidence intervals were obtained using a multivariable Cox model including gender, race, baseline age group (≤ 37 or >37 at baseline), study cohort and estimated CD4 slope and intercept.

Second, joint modeling approach that enables both longitudinal CD4 measurements and clinical endpoint data to be modelled together while accounting for the association between the corresponding linear mixed-effects and Cox proportional hazards models was used.(1, 2) Specifically, the joint model combines the mixed effects linear random effects submodel for the repeated CD4 count measurements and the time to event Cox submodel for the composite outcome so that the effect of the longitudinal CD4 measurements can be separated from its effect on the survival process. In this way, joint modeling provides less biased estimations and more efficient inferences than the two-stage approach.

The family of joint models is also called the “shared parameter models” (SPMs).

**Comparison of CD4 count metrics.**

Time-dependent receiver operator characteristic (ROC) curves, the time-dependent area under the curves (AUC) and Uno’s C-statistics were used to assess and compare the discriminative performance of the final prediction model. All three statistics were appropriate for right-censored data. These three statistics were used to characterize the fit of the Cox model and determine how well the model distinguished between patients with and without the composite endpoint. Time-dependent ROC curves were generated at 5-years and 10-years after initiation of ART. Time-dependent AUCs and Uno’s C-statistics were also computed over the entire study period and therefore provides an overall summary measure of predictive accuracy. The inverse probability of censoring weighting approach was adopted to compute time-dependent ROC curves and time-dependent AUC statistics.

**References**

1. Wulfsohn MS, Tsiatis AA. A joint model for survival and longitudinal data measured with error. Biometrics. 1997;53(1):330-9.

2. Tsiatis AA, DeGruttola V, MW W. Modeling the relationship of survivial to longitudinal data measured with error. Applications to survival and CD4 counts in patients with AIDS. Journal of the American Statistical Association. 1995;90:22-37.
