# Supplementary material for: CD4 rate of increase is preferred to CD4 threshold for predicting outcomes among virologically suppressed HIV-infected adults on antiretroviral therapy
Source: PLoS One. 2020 Jan 6;15(1):e0227124. doi: 10.1371/journal.pone.0227124 (PMC6944336; doi:10.1371/journal.pone.0227124)
Supplement: S2 Appendix — (DOCX) [file pone.0227124.s002.docx]

**S2 Appendix**

**SAS v9.4 code for conducting two-stage modeling and joint modeling analyses(1)**

In the SAS code below ds_l represents a dataset containing the longitudinal CD4 cell counts over the first two years (cd4_count), time points at which CD4 cell counts were measured (cd4_year) and patient indicator (mrn_ID). ds_c represents a dataset containing the follow-up time (followup), composite event indicator (event), patient indicator (mrn_ID) and covariates. For this code, variables *var1* (continuous) and *var2* (dichotomous) are potential confounders measured at baseline.

***** Two-Stage Modeling*****;

/* Linear mixed effects model (first stage) with random intercept and random slope fitted on the longitudinal CD4 counts by time. */

/* The output dataset sf contains fixed intercept and slope estimates, and another output dataset sr contains random intercept and slope estimates. */

proc mixed method=ml data=ds_l;

class mrn_ID;

model cd4_count=cd4_year/solution;

random int cd4_year /type=un subject=mrn_ID solution;

ods output solutionf=sf(keep=effect estimate

rename=(estimate=overall));

ods output solutionr=sr(keep= effect mrn_ID estimate

rename=(estimate=individual));

run;

/* Compute patient-specific CD4 cell count intercept and slope estimates using the corresponding fixed and random effect estimates. */

/* The dataset linearcd4 contains two variables: (1) the variable individual_cd4_intercept is the patient-specific CD4 cell count intercept estimate; and (2) the variable individual_cd4_slope is the patient-specific CD4 cell count slope estimate*/

proc sort data=sf;

by effect;

run;

proc sort data=sr;

by effect;

run;

data final;

merge sf sr;

by effect;

patient_cd4_slope = overall + individual;

run;

proc sort data=final;

by mrn_ID effect;

run;

data linearcd4;

set final;

by mrn_ID;

patient_cd4_intercept=lag1(patient_cd4_slope);

if Effect="cd4_year";

drop Effect overall individual;

run;

/* Merge datasets ds_c and linearcd4, and create a new dataset ds. */

data ds;

merge ds_c linearcd4;

by mrn_ID;

run;

/* Evaluate association between the estimated patient-level CD4 cell count slope (and intercept) and the composite endpoint using the Cox PH model (second stage). */

/* Adjusted PH model */

proc phreg data = ds;

class var2;

model followup*event(0)=patient_cd4_intercept patient_cd4_slope var1 var2;

run;

***** Joint Modeling*****;

/* Call JMFit Macro in order to fit the joint model. */

filename jmfit "C:\directory";

%include jmfit(JMFit.sas);

%*JMFit*(LONG=, SURV=, MODEL=, TS=, TMAXI=, WEIGHT=, NPIECES=, PARTITION=, OPTIONS=, INITIAL=, OUTPUT=);

/* Run the joint model. */

/* Long represents a dataset that contains longitudinal CD4 measurements, and SURV represents a dataset that contains survival outcome. SPM2L (Shared Parameter Model 2 with Linear trajectory) in the Model statement represents a joint model that consists of linear random effects submodel and a Cox PH submodel with beta parameters for each random effect.*/

%*JMFit*(LONG=ds_l, SURV=ds_c, MODEL=SPM2L, TS=0, NPIECES=1,PARTITION=1) ;

**References**

1. Zhang D, Chen MH, Ibrahim JG, Boye ME, Shen W. JMFit: A SAS Macro for Joint Models of Longitudinal and Survival Data. J Stat Softw. 2016;71(3).
