# Supplementary material for: CD4 rate of increase is preferred to CD4 threshold for predicting outcomes among virologically suppressed HIV-infected adults on antiretroviral therapy
Source: PLoS One. 2020 Jan 6;15(1):e0227124. doi: 10.1371/journal.pone.0227124 (PMC6944336; doi:10.1371/journal.pone.0227124)
Supplement: S3 Appendix — (DOCX) [file pone.0227124.s003.docx]

**S3 Appendix**

**INR definitions in the literature**

| Author/journal | Cohort details | Time Period | CD4 Metric/INR definition |
| --- | --- | --- | --- |
| CD4 Threshold | | | |
| Cenderello et al, *Expert Rev Anti Infect Ther*, 2016(1) | None used | 18-36 months | PR: <500 threshold + >200 cell increase  INR: <500 threshold and <200 cell increase |
| Engsig et al, *CID*, 2014(2) | ART-CC, COHERE cohort,  CD4 at start <200 | 3 years | CD4 threshold < 200 cell/μL |
| Kaufmann et al, *CID*, 2005(3) | Swiss HIV Cohort | 5 years | CD4 threshold < 500 cell/μL |
| Lederman et al, *JID*, 2011(4) | CD4 at start <350 | 2 years | IR: >500 threshold  INR: <350 threshold |
| Loufty et al, *JAIDS*, 2010(5) | CANOC cohort | 1 or 2 years | CD4 threshold <200 cell/μL |
| Pacheco et al, *Antiviral Res*, 2015(6) | CoRIS Cohort  CD4 at start <200 | 2 years | CD4 threshold < 250 cell/μL |
| Falster et al, *J Acquir Immune Defic Syndr,* 2009(7) | AHOD  CD4 baseline <350 | 9-24 months | CD4 threshold <350 cell/μL |
| CD4/CD8 ratio | | | |
| Castilho et al, *AIDS*, 2016(8) | Vanderbilt Cohort | 12 months after viral suppression | CD4/CD8 ratio <0.4 |
| CD4 increase | | | |
| Batista et al, *Med Mal Infect*, 2015(9) | Dakar ATC cohort | 6 months | CD4 increase <50 cell/μL |
| Gilson et al, *HIV medicine*, 2010(10) | UK CHIC study | 6-10 months, 10-14 months | CD4 increase < 100 cell/μL at two-time points |
| Grabar et al, *Ann Inter Med*, 2000(11) | French Hospital Database on HIV | 6 months | CD4 increase <50 cell/μL |
| Baker et al, *J Acquir Immune Defic Syndr* 2008(12) | FIRST trial | 8 months | CD4 increase <50 cell/μL |
| Nicastri et al, *J Med Virol*, 2005(13) | IATG | 12 months | CD4 increase ≤ 100 cell/μL |
| Tan et al, *J Acquir Immune Defic Syndr*, 2008(14)  Tuboi et al, *J Acquir Immune Defic Syndr*, 2010(15)  Moore et al, *J Acquir Immune Defic Syndr,*2005(16) | UAB 1917 Clinic  ART-LINC of IeDEA  HIV/AIDS DTP of the British Columbia Centre for Excellence | 3-9 months | CD4 increase <50 cell/μL |

**References**

1. Cenderello G, De Maria A. Discordant responses to cART in HIV-1 patients in the era of high potency antiretroviral drugs: clinical evaluation, classification, management prospects. Expert Rev Anti Infect Ther. 2016;14(1):29-40.

2. Engsig FN, Zangerle R, Katsarou O, Dabis F, Reiss P, Gill J, et al. Long-term mortality in HIV-positive individuals virally suppressed for >3 years with incomplete CD4 recovery. Clin Infect Dis. 2014;58(9):1312-21.

3. Kaufmann GR, Furrer H, Ledergerber B, Perrin L, Opravil M, Vernazza P, et al. Characteristics, determinants, and clinical relevance of CD4 T cell recovery to <500 cells/microL in HIV type 1-infected individuals receiving potent antiretroviral therapy. Clin Infect Dis. 2005;41(3):361-72.

4. Lederman MM, Calabrese L, Funderburg NT, Clagett B, Medvik K, Bonilla H, et al. Immunologic failure despite suppressive antiretroviral therapy is related to activation and turnover of memory CD4 cells. J Infect Dis. 2011;204(8):1217-26.

5. Loutfy MR, Genebat M, Moore D, Raboud J, Chan K, Antoniou T, et al. A CD4+ cell count <200 cells per cubic millimeter at 2 years after initiation of combination antiretroviral therapy is associated with increased mortality in HIV-infected individuals with viral suppression. J Acquir Immune Defic Syndr. 2010;55(4):451-9.

6. Pacheco YM, Jarrin I, Rosado I, Campins AA, Berenguer J, Iribarren JA, et al. Increased risk of non-AIDS-related events in HIV subjects with persistent low CD4 counts despite cART in the CoRIS cohort. Antiviral Res. 2015;117:69-74.

7. Falster K, Petoumenos K, Chuah J, Mijch A, Mulhall B, Kelly M, et al. Poor baseline immune function predicts an incomplete immune response to combination antiretroviral treatment despite sustained viral suppression. J Acquir Immune Defic Syndr. 2009;50(3):307-13.

8. Castilho JL, Shepherd BE, Koethe J, Turner M, Bebawy S, Logan J, et al. CD4+/CD8+ ratio, age, and risk of serious noncommunicable diseases in HIV-infected adults on antiretroviral therapy. AIDS. 2016;30(6):899-908.

9. Batista G, Buve A, Ngom Gueye NF, Manga NM, Diop MN, Ndiaye K, et al. Initial suboptimal CD4 reconstitution with antiretroviral therapy despite full viral suppression in a cohort of HIV-infected patients in Senegal. Med Mal Infect. 2015;45(6):199-206.

10. Gilson RJ, Man SL, Copas A, Rider A, Forsyth S, Hill T, et al. Discordant responses on starting highly active antiretroviral therapy: suboptimal CD4 increases despite early viral suppression in the UK Collaborative HIV Cohort (UK CHIC) Study. HIV Med. 2010;11(2):152-60.

11. Grabar S, Le Moing V, Goujard C, Leport C, Kazatchkine MD, Costagliola D, et al. Clinical outcome of patients with HIV-1 infection according to immunologic and virologic response after 6 months of highly active antiretroviral therapy. Ann Intern Med. 2000;133(6):401-10.

12. Baker JV, Peng G, Rapkin J, Krason D, Reilly C, Cavert WP, et al. Poor initial CD4+ recovery with antiretroviral therapy prolongs immune depletion and increases risk for AIDS and non-AIDS diseases. J Acquir Immune Defic Syndr. 2008;48(5):541-6.

13. Nicastri E, Chiesi A, Angeletti C, Sarmati L, Palmisano L, Geraci A, et al. Clinical outcome after 4 years follow-up of HIV-seropositive subjects with incomplete virologic or immunologic response to HAART. J Med Virol. 2005;76(2):153-60.

14. Tan R, Westfall AO, Willig JH, Mugavero MJ, Saag MS, Kaslow RA, et al. Clinical outcome of HIV-infected antiretroviral-naive patients with discordant immunologic and virologic responses to highly active antiretroviral therapy. J Acquir Immune Defic Syndr. 2008;47(5):553-8.

15. Tuboi SH, Pacheco AG, Harrison LH, Stone RA, May M, Brinkhof MW, et al. Mortality associated with discordant responses to antiretroviral therapy in resource-constrained settings. J Acquir Immune Defic Syndr. 2010;53(1):70-7.

16. Moore DM, Hogg RS, Yip B, Wood E, Tyndall M, Braitstein P, et al. Discordant immunologic and virologic responses to highly active antiretroviral therapy are associated with increased mortality and poor adherence to therapy. J Acquir Immune Defic Syndr. 2005;40(3):288-93.
