# Supplementary material for: CD4 rate of increase is preferred to CD4 threshold for predicting outcomes among virologically suppressed HIV-infected adults on antiretroviral therapy
Source: PLoS One. 2020 Jan 6;15(1):e0227124. doi: 10.1371/journal.pone.0227124 (PMC6944336; doi:10.1371/journal.pone.0227124)
Supplement: S1 Table — (DOCX) [file pone.0227124.s004.docx]

**S1 Table. Estimated^a^ mean CD4/CD8 ratio intercept, estimated mean CD4/CD8 ratio after 1 and 2 years from ART initiation and the estimated mean CD4/CD8 ratio slope (per year) during the two years following ART initiation with treatment interruptions of less than 2 months by cohort, CD4/CD8 ratio recovery status and baseline age group.**

|  | Mean CD4/CD8 Intercept (SE) | Mean CD4/CD8 slope (SE) | Mean CD4/CD8 Ratio at Year 1 (SE) | Mean CD4/CD8 Ratio at Year 2 (SE) |
| --- | --- | --- | --- | --- |
| All | 0.432 (0.007) | 0.169 (0.003) | 0.601 (0.008) | 0.770 (0.010) |
| Cohort^b^ |  |  |  |  |
| AVAMC | 0.444 (0.018) | 0.162 (0.010) | 0.607 (0.020) | 0.768 (0.026) |
| IDP | 0.248 (0.009) | 0.151 (0.005) | 0.399 (0.010) | 0.550 (0.013) |
| NHS | 0.632 (0.009) | 0.189 (0.005) | 0.820 (0.010) | 1.009 (0.013) |
| P-value^g^ | < 0.0001 | < 0.0001 | < 0.0001 | < 0.0001 |
| CD4/CD8 recovery status^c^ |  |  |  |  |
| Non-responders | 0.329 (0.009) | 0.066 (0.003) | 0.395 (0.009) | 0.461 (0.011) |
| Responders | 0.533 (0.010) | 0.274 (0.004) | 0.807 (0.009) | 1.080 (0.011) |
| P-value^g^ | < 0.0001 | < 0.0001 | < 0.0001 | < 0.0001 |
| Baseline age group^d^ |  |  |  |  |
| Age > 37 | 0.360 (0.010) | 0.154 (0.005) | 0.514 (0.011) | 0.667 (0.014) |
| Age ≤37 | 0.497 (0.010) | 0.183 (0.005) | 0.680 (0.011) | 0.863 (0.013) |
| P-value^g^ | < 0.0001 | < 0.0001 | < 0.0001 | < 0.0001 |
| Race^e^ |  |  |  |  |
| Caucasian | 0.524 (0.014) | 0.170 (0.007) | 0.693 (0.017) | 0.863 (0.021) |
| African-American | 0.385 (0.009) | 0.168 (0.004) | 0.553 (0.009) | 0.721 (0.012) |
| Hispanic | 0.473 (0.026) | 0.170 (0.010) | 0.642 (0.028) | 0.812 (0.034) |
| Other/not specified | 0.494 (0.040) | 0.178 (0.015) | 0.672 (0.047) | 0.849 (0.057) |
| P-value^g^ | < 0.0001 | 0.9439 | < 0.0001 | < 0.0001 |
| Gender^f^ |  |  |  |  |
| Female | 0.356 (0.018) | 0.199 (0.009) | 0.554 (0.020) | 0.753 (0.025) |
| Male | 0.443 (0.008) | 0.165 (0.004) | 0.608 (0.009) | 0.772 (0.011) |
| P-value^g^ | < 0.0001 | 0.005 | 0.0137 | 0.4793 |

Abbreviations: SE= standard error. AVAMC= Infectious Disease Clinic at the Atlanta Veterans Affairs (VA) Medical Center. IDP= Infectious Disease Program of the Grady Health System. NHS= US Military HIV Natural History Study

^a^Estimated from linear mixed-effects model specifying that CD4/CD8 ratio during the two years following ART initiation follow a linear model regression over time, with a random intercept and slope for each patient.

^b^Additional categorical covariate representing the study cohort is included the linear mixed-effects model. ^c^Additional binary covariate representing the immune status is included the linear mixed-effects model.

^d^Additional binary covariate representing the baseline age group is included the linear mixed-effects model.

^e^Additional categorical covariate representing the race group is included the linear mixed-effects model.

^f^Additional binary covariate representing the gender is included the linear mixed-effects model. ^g^Result of a likelihood ratio test comparing estimated CD4/CD8 ratio intercepts, counts at years 1 and 2, and CD4/CD8 ratio slopes among different categories of a given covariate.
