# Supplementary material for: CD4 rate of increase is preferred to CD4 threshold for predicting outcomes among virologically suppressed HIV-infected adults on antiretroviral therapy
Source: PLoS One. 2020 Jan 6;15(1):e0227124. doi: 10.1371/journal.pone.0227124 (PMC6944336; doi:10.1371/journal.pone.0227124)
Supplement: S2 Table — (DOCX) [file pone.0227124.s005.docx]

**S2 Table: Association of estimated CD4/CD8 ratio slope and intercept during the two years following ART initiation with risk of composite endpoint^a^**

|  | Unadjusted^b^ | | |  | Adjusted^c^ | | |
| --- | --- | --- | --- | --- | --- | --- | --- |
|  | HR | 95% CI | P-value |  | HR | 95% CI | P-value |
| Two-Stage Model^a^ |  |  |  |  |  |  |  |
| Estimated CD4/CD8 Intercept |  |  | 0.0004 |  |  |  | 0.0821 |
| Per 0.1 increase | 0.94 | 0.91 – 0.98 |  |  | 0.97 | 0.93 – 1.01 |  |
| Per 0.2 increase | 0.89 | 0.84 – 0.95 |  |  | 0.94 | 0.87 – 1.01 |  |
| Regression coefficient | $\hat{\beta}$ (SE) = -0.6045 (0.2455) | | |  | $\hat{\beta}$ (SE) = -0.2950 (0.2623) | | |
| Estimated CD4/CD8 Slope |  |  | < 0.0001 |  |  |  | 0.0001 |
| 0.1 higher rate per year | 0.70 | 0.60 – 0.83 |  |  | 0.74 | 0.63 – 0.86 |  |
| 0.2 higher rate per year | 0.50 | 0.36 – 0.69 |  |  | 0·55 | 0.40 – 0.74 |  |
| Regression coefficient | $\hat{\beta}$ (SE) = -3.5103 (0.8339) | | |  | $\hat{\beta}$ (SE) = -3.0294 (0.7927) | | |
| Joint Model^d^ |  |  |  |  |  |  |  |
| Estimated CD4/CD8 Intercept |  |  | 0.0293 |  |  |  | 0.1959 |
| Per 0.1 increase | 0.95 | 0.90 – 1.03 |  |  | 0.97 | 0.92 – 1.02 |  |
| Per 0.2 increase | 0.90 | 0.82 – 0.99 |  |  | 0.93 | 0.84 – 1.04 |  |
| Regression coefficient | $\hat{\beta}$ (SE) = -0.5189 (0.2426) | | |  | $\hat{\beta}$ (SE) = -0.2939 (0.2661) | | |
| Estimated CD4/CD8 Slope |  |  | < 0.0001 |  |  |  | 0.0003 |
| 0.1 higher rate per year | 0.71 | 0.61 – 0.84 |  |  | 0.75 | 0.64 – 0.88 |  |
| 0.2 higher rate per year | 0.51 | 0.37 – 0.71 |  |  | 0.56 | 0.41 – 0.76 |  |
| Regression coefficient | $\hat{\beta}$ (SE) = -3.3642 (0.8359) | | |  | $\hat{\beta}$ (SE) = -2.9078 (0.7990) | | |

^a^The risk of composite endpoint was modeled using a Cox proportional hazards regression model considering estimated CD4/CD8 ratio slope and intercept obtained from the linear mixed-effects model as continuous covariates.

^b^Results obtained from Cox submodels for the composite outcome that include estimated CD4/CD8 ratio slope and intercept as covariates.

^c^Results obtained from the Cox submodels for the composite outcome that include estimated CD4/CD8 ratio slope and intercept, study cohort, and baseline age group ($\leq$ 37 or >37 at baseline) as covariates.

^d^The risk of composite endpoint was modeled using the joint model that enables both longitudinal CD4/CD8 ratio measurements and clinical endpoint data to be modelled together while taking account for the interrelationship between the two components.

Abbreviations: CI= confidence interval. HR= hazard ratio.
