# Supplementary material for: CD4 rate of increase is preferred to CD4 threshold for predicting outcomes among virologically suppressed HIV-infected adults on antiretroviral therapy
Source: PLoS One. 2020 Jan 6;15(1):e0227124. doi: 10.1371/journal.pone.0227124 (PMC6944336; doi:10.1371/journal.pone.0227124)
Supplement: S3 Table — (DOCX) [file pone.0227124.s006.docx]

**S3 Table. Association between patient CD4 recovery status based on estimated CD4 slope and risk of composite endpoint by baseline age group, gender and race**

| CD4 Recovery Status^a^ | Effect Modification^b^ | HR | 95% CI | P-value |
| --- | --- | --- | --- | --- |
| Immune Responders  (Estimated CD4 Slope $\boldsymbol{>}$ 100)  vs. Immune Non-responders  (Estimated CD4 Slope $\boldsymbol{\leq}$ 100) | - | 0.73 | 0.56 – 0.94 | 0.0132 |
| Immune Responders  (Estimated CD4 Slope $\boldsymbol{>}$ 100)  vs. Immune Non-responders  (Estimated CD4 Slope $\boldsymbol{\leq}$ 100) | Age > 37 | 0.61 | 0.44 – 0.84 | 0.0027 |
|  | Age $\leq$ 37 | 0.98 | 0.65 – 1.49 | 0.9411 |
| Immune Responders  (Estimated CD4 Slope $\boldsymbol{>}$ 100)  vs. Immune Non-responders  (Estimated CD4 Slope $\boldsymbol{\leq}$ 100) | Female | 0.57 | 0.26 – 1.23 | 0.1519 |
|  | Male | 0.75 | 0.57 – 0.98 | 0.0330 |
| Immune Responders  (Estimated CD4 Slope $\boldsymbol{>}$ 100)  vs. Immune Non-responders  (Estimated CD4 Slope $\boldsymbol{\leq}$ 100) | Caucasian | 0.74 | 0.48 – 1.14 | 0.1739 |
|  | African American | 0.70 | 0.52 – 0.94 | 0.0163 |
|  | Hispanic | 1.24 | 0.63 – 2.45 | 0.5384 |
|  | Other | 0.31 | 0.04 – 2.19 | 0.2376 |

^a^The risk of composite endpoint was modeled using a multivariate Cox proportional hazards regression model that includes a binary covariate representing patient CD4 recovery status (immune responders vs. immune non-responders) based on the estimated CD4 slope obtained from the linear mixed-effect model. Immune non-responders is the reference group.

^b^Effect modification is defined in terms of the effect on CD4 recovery status varying across strata of a second variable (i.e. formally tested as an interaction between CD4 recovery status and second variable). Effect modification by baseline age group and gender was assessed using a multivariate Cox proportional hazards model adjusted for CD4 recovery status, estimated CD4 intercept, study cohort, baseline age group ($\leq$ 37 or >37 at baseline) and gender. Effect modification by race was assessed using a multivariate Cox proportional hazards model adjusted for CD4 recovery status, estimated CD4 intercept, study cohort, baseline age group ($\leq$ 37 or >37 at baseline), gender and race.

Abbreviations: CI= confidence interval. HR= hazard ratio.
