# Supplementary material for: CD4 rate of increase is preferred to CD4 threshold for predicting outcomes among virologically suppressed HIV-infected adults on antiretroviral therapy
Source: PLoS One. 2020 Jan 6;15(1):e0227124. doi: 10.1371/journal.pone.0227124 (PMC6944336; doi:10.1371/journal.pone.0227124)
Supplement: S4 Table — (DOCX) [file pone.0227124.s007.docx]

**S4 Table. Association between patient CD4/CD8 ratio recovery status based on estimated CD4/CD8 ratio slope and risk of composite endpoint by baseline age group, gender and race.**

| CD4/CD8 Recovery Status^a^ | Effect Modification^b^ | HR | 95% CI | P-value |
| --- | --- | --- | --- | --- |
| Immune Responders  (Estimated CD4/CD8 Slope $\boldsymbol{>}$ 0.15)  vs. Immune Non-responders  (Estimated CD4/CD8 Slope $\boldsymbol{\leq}$ 0.15) | - | 0.64 | 0.48 – 0.84 | 0.0015 |
| Immune Responders  (Estimated CD4/CD8 Slope $\boldsymbol{>}$ 0.15)  vs. Immune Non-responders  (Estimated CD4/CD8 Slope $\boldsymbol{\leq}$ 0.15) | Age > 37 | 0.50 | 0.35 – 0.72 | 0.0002 |
|  | Age $\leq$ 37 | 0.91 | 0.59 – 1.39 | 0.6650 |
| Immune Responders  (Estimated CD4/CD8 Slope $\boldsymbol{>}$ 0.15)  vs. Immune Non-responders  (Estimated CD4/CD8 Slope $\boldsymbol{\leq}$ 0.15) | Female | 0.47 | 0.21 – 1.05 | 0.0662 |
|  | Male | 0.68 | 0.51 – 0.91 | 0.0105 |
| Immune Responders  (Estimated CD4/CD8 Slope $\boldsymbol{>}$ 0.15)  vs. Immune Non-responders  (Estimated CD4/CD8 Slope $\boldsymbol{\leq}$ 0.15) | Caucasian | 0.73 | 0.47 – 1.13 | 0.1546 |
|  | African American | 0.60 | 0.43 – 0.82 | 0.0016 |
|  | Hispanic | 0.82 | 0.39 – 1.69 | 0.5860 |
|  | Other | 0.49 | 0.12 – 1.99 | 0.3184 |

^a^The risk of composite endpoint was modeled using a multivariate Cox proportional hazards regression model that includes a binary covariate representing patient CD4/CD8 ratio recovery status (immune responders vs. immune non-responders) based on the estimated CD4/CD8 ratio slope obtained from the linear mixed-effect model. Immune non-responders is the reference group.

^b^Effect modification by baseline age group was assessed using a multivariate Cox proportional hazards model adjusted for CD4/CD8 ratio recovery status, estimated CD4/CD8 ratio intercept, study cohort, and baseline age group ($\leq$ 37 or >37 at baseline). Effect modification by gender was assessed using a multivariate Cox proportional hazards model adjusted for CD4/CD8 ratio recovery status, estimated CD4/CD8 ratio intercept, study cohort, baseline age group ($\leq$ 37 or >37 at baseline) and gender. Effect modification by race was assessed using a multivariate Cox proportional hazards model adjusted for CD4/CD8 ratio recovery status, estimated CD4/CD8 ratio intercept, study cohort, baseline age group ($\leq$ 37 or >37 at baseline), and race.

Abbreviations: CI= confidence interval; HR= hazard ratio.
