# Supplementary material for: CD4 rate of increase is preferred to CD4 threshold for predicting outcomes among virologically suppressed HIV-infected adults on antiretroviral therapy
Source: PLoS One. 2020 Jan 6;15(1):e0227124. doi: 10.1371/journal.pone.0227124 (PMC6944336; doi:10.1371/journal.pone.0227124)
Supplement: S5 Table — (DOCX) [file pone.0227124.s008.docx]

**S5 Table. Comparison of Uno’s C-statistics among the four prognostic risk factors: (i) Estimated CD4 slope (ii) Estimated CD4/CD8 ratio slope (iii) CD4 counts after 1 year and (iv) CD4 counts after 2 years**

| Prognostic Risk Factors | Uno’s C-statistics (SE) | P-value |
| --- | --- | --- |
| Unadjusted^a^ |  |  |
| Estimated CD4 Slope  vs. Estimated CD4/CD8 Slope | 0.5622 (0.0204) vs. 0.6187 (0.0223) | **0**.**0072** |
| Estimated CD4 Slope  vs. CD4 count at 1 year | 0.5622 (0.0204) vs. 0.5724 (0.0224) | 0.3054 |
| Estimated CD4 Slope  vs. CD4 count at 2 years | 0.5622 (0.0204) vs. 0.5690 (0.0232) | 0.5496 |
| Estimated CD4/CD8 Slope  vs. CD4 count at 1 year | 0.6187 (0.0223) vs. 0.5724 (0.0224) | **0**.**0328** |
| Estimated CD4/CD8 Slope  vs. CD4 count at 2 years | 0.6187 (0.0223) vs. 0.5690 (0.0232) | **0**.**0307** |
| CD4 count at 1 year  vs. CD4 count at 2 years | 0.5724 (0.0224) vs. 0.5690 (0.0232) | 0.8044 |
| Adjusted^b^ |  |  |
| Estimated CD4 Slope  vs. Estimated CD4/CD8 Slope | 0.6456 (0.0201) vs. 0.6604 (0.0206) | 0.1353 |
| Estimated CD4 Slope  vs. CD4 count at 1 year | 0.6456 (0.0201) vs. 0.6445 (0.0203) | 0.8089 |
| Estimated CD4 Slope  vs. CD4 count at 2 years | 0.6456 (0.0201) vs. 0.6415 (0.0191) | 0.5083 |
| Estimated CD4/CD8 Slope  vs. CD4 count at 1 year | 0.6604 (0.0206) vs. 0.6445 (0.0203) | 0.0899 |
| Estimated CD4/CD8 Slope  vs. CD4 count at 2 years | 0.6604 (0.0206) vs. 0.6415 (0.0191) | 0.0645 |
| CD4 count at 1 year  vs. CD4 count at 2 years | 0.6445 (0.0203) vs. 0.6415 (0.0191) | 0.6039 |

^a^Cox regression models containing each one of estimated CD4 slope, estimated CD4/CD8 ratio, CD4 cell count at 1 year and CD4 cell count at 2 years as a main predictor and adjusted for each one of estimated CD4 intercept, estimated CD4/CD8 ratio intercept and baseline CD4 count (for last two factors).

^b^Cox regression models containing each one of estimated CD4 slope, estimated CD4/CD8 ratio, CD4 cell count at 1 year and CD4 cell count at 2 years as a main predictor and adjusted for each one of estimated CD4 intercept, estimated CD4/CD8 ratio intercept and baseline CD4 count, and commonly adjusted for study cohort, age at baseline and gender
