# Supplementary figures and images for: CD4 rate of increase is preferred to CD4 threshold for predicting outcomes among virologically suppressed HIV-infected adults on antiretroviral therapy
Source: PLoS One. 2020 Jan 6;15(1):e0227124. doi: 10.1371/journal.pone.0227124 (PMC6944336; doi:10.1371/journal.pone.0227124)

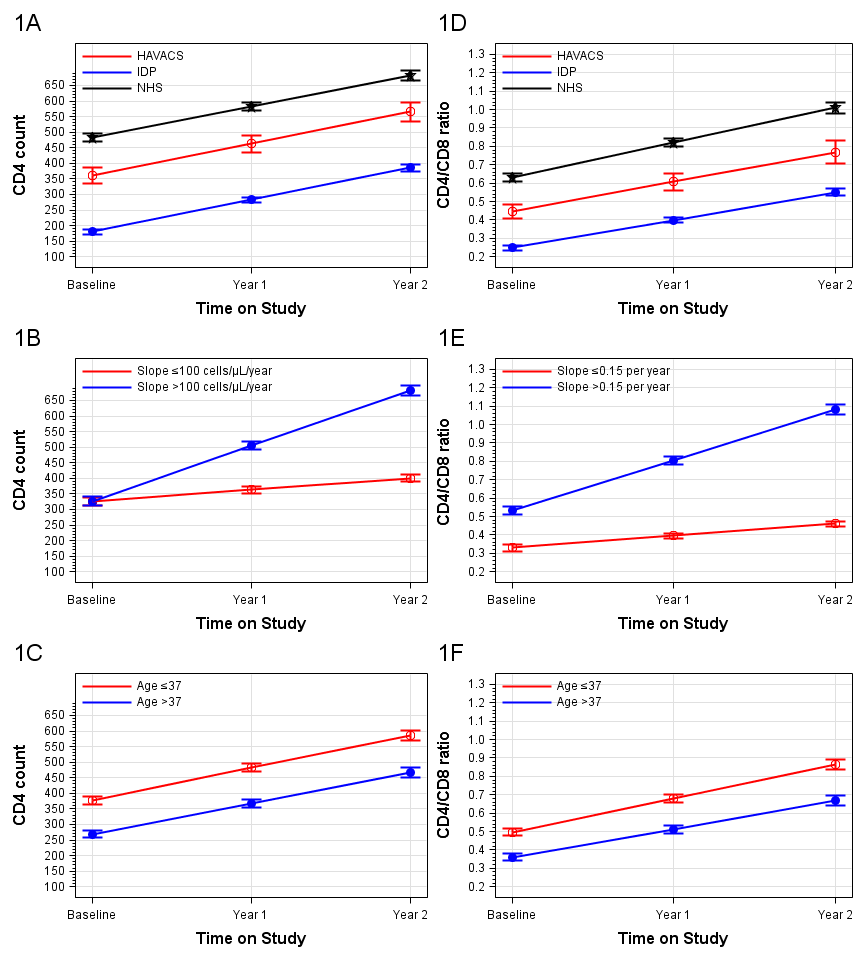

Supplement: S1 Fig — Estimated mean CD4 cell counts (cells/ μL) (left panel) and estimated mean CD4/CD8 ratio (right panel) at baseline and after 1 and 2 years in the first 2 years on continuous ART for at least 24 months by study cohort (S1a, S1d), immune status (S1b, S1e) and age at baseline (S1c, S1f). Vertical bars are 95% confidence intervals. (TIF) [file pone.0227124.s009.tif]

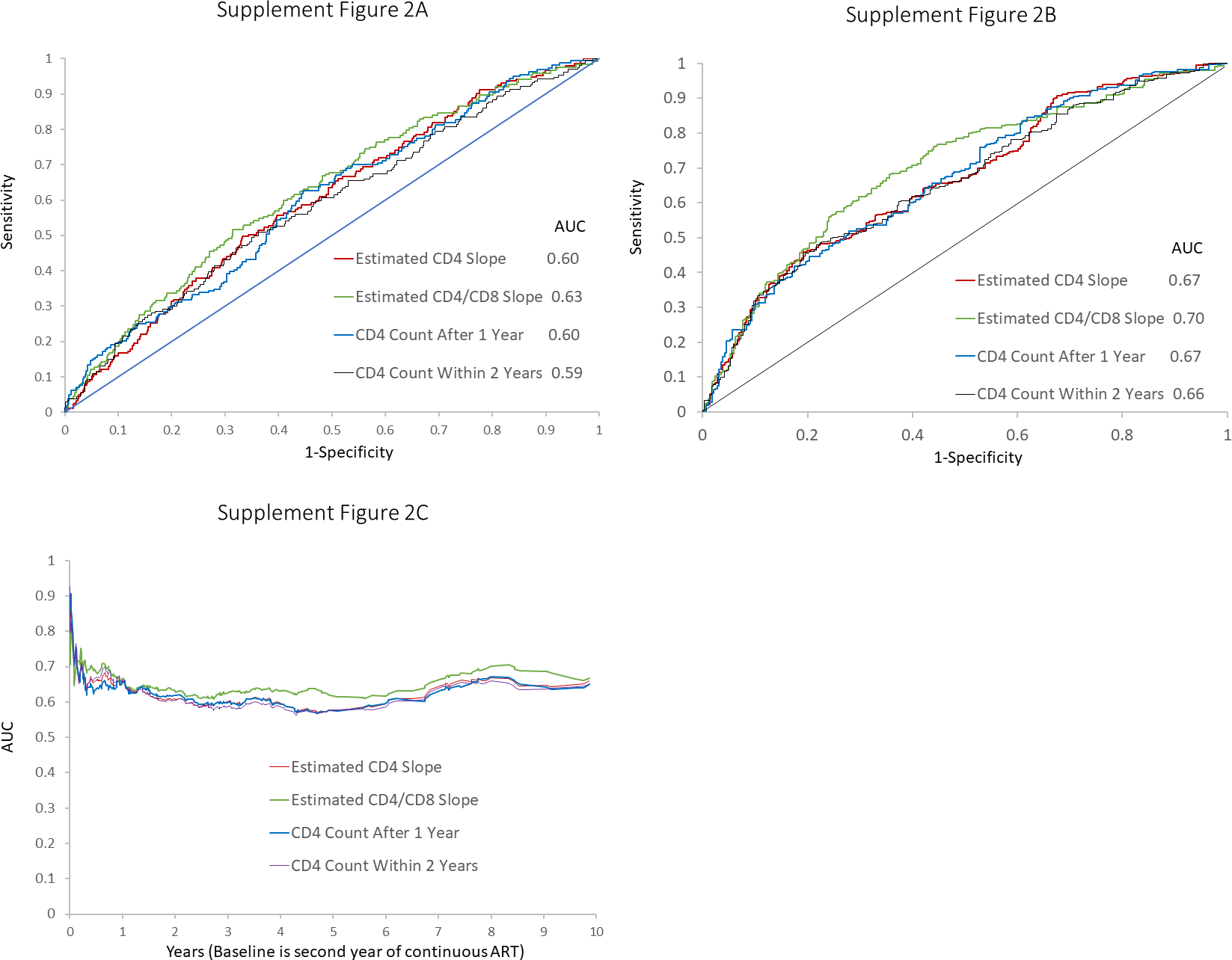

Supplement: S2 Fig — (a) CD4 metrics time-dependent ROC curves at 5 years from ART initiation. (b) CD4 metrics time-dependent ROC curves at 10 years from ART initiation. (c) CD4 metrics time-dependent AUCs evaluated over the 10-year study period. (TIF) [file pone.0227124.s010.tif]
